# Examining the contribution of motor movement and language dominance to increased left lateralization during sign generation in native signers

**DOI:** 10.1016/j.bandl.2016.06.004

**Published:** 2016-08

**Authors:** Eva Gutierrez-Sigut, Heather Payne, Mairéad MacSweeney

**Affiliations:** a Deafness, Cognition & Language Research Centre, University College London, United Kingdom; b Departamento de Metodología de las Ciencias del Comportamiento, Universitat de València, Spain; c Institute of Cognitive Neuroscience, University College London, United Kingdom

**Keywords:** fTCD, Language lateralization, Overt language production, Sign language, Phonological fluency, Semantic fluency

## Abstract

The neural systems supporting speech and sign processing are very similar, although not identical. In a previous fTCD study of hearing native signers (Gutierrez-Sigut, Daws, et al., 2015) we found stronger left lateralization for sign than speech. Given that this increased lateralization could not be explained by hand movement alone, the contribution of motor movement versus ‘linguistic’ processes to the strength of hemispheric lateralization during sign production remains unclear. Here we directly contrast lateralization strength of covert versus overt signing during phonological and semantic fluency tasks. To address the possibility that hearing native signers’ elevated lateralization indices (LIs) were due to performing a task in their less dominant language, here we test deaf native signers, whose dominant language is British Sign Language (BSL). Signers were more strongly left lateralized for overt than covert sign generation. However, the strength of lateralization was not correlated with the amount of time producing movements of the right hand. Comparisons with previous data from hearing native English speakers suggest stronger laterality indices for sign than speech in both covert and overt tasks. This increased left lateralization may be driven by specific properties of sign production such as the increased use of self-monitoring mechanisms or the nature of phonological encoding of signs.

## Highlights

• We tested hemispheric lateralization for language in deaf native signers.
• Signers were more strongly left lateralized for overt than covert sign generation.
• We found stronger left lateralization for BSL than for English production.
• Stronger left lateralization for BSL is not driven by motoric activity alone.
• Stronger left lateralization is not driven by language dominance.

## 1 Introduction

In a recent study of hemispheric lateralization of language production in hearing native signers we showed stronger left lateralization for British Sign Language (BSL) than for speech during overt sign and word generation tasks (Gutierrez-Sigut, Daws, et al., 2015). Sign production requires predominantly asymmetrical movements of the arms and hands (Battison, 1978), yet strength of lateralization during sign production did not show a robust correlation with amount of time producing movements of the right hand. In addition, hearing native signers showed much stronger lateralization during BSL production than hearing non-signers who performed a non-sign repetition task. Together these findings suggest that the stronger left lateralization found during sign production in native signers could not wholly be explained by movement of the right hand and may also be due to specific sign processing factors.

Unlike phonological encoding of words, which requires the selection and arrangement in time of a series of phonemes, phonological encoding of signs requires the selection of a particular handshape in a specific body location and a movement (see e.g. Stokoe, 1960). Furthermore, while the speaker can directly hear her own utterances, the signer has only partial perceptual feedback of her own signing. Even when she can see her hands moving in space, her point of view is different to that during sign perception. This raises the likelihood that in order to keep track of the position and precise movements of the hands, overt sign production relies more on proprioceptive and somatosensory feedback than speech. These factors have been linked with increased left parietal activation found in previous neuroimaging studies (Corina et al., 2003, Emmorey et al., 2014, Emmorey et al., 2007). It is also possible however that the stronger lateralization is due to two other factors, not wholly addressed in our previous study: the precise role of motor movement, and the language dominance status of the hearing signers.

In our previous study of sign and speech production, we assessed the contribution of motor movement to strength of lateralization by examining correlations with the amount of hand movement (Gutierrez-Sigut, Daws, et al., 2015). Using this correlational approach, we showed no influence of amount of motor movement on strength of lateralization indices (LIs) when participants performed a BSL semantic fluency task. A moderate correlation was found for the BSL phonological fluency task, which we suggested could relate to the motoric prompting strategy used by participants when presented with a phonological target (handshape). Participants tended to maintain the target handshape, moving it to different locations in an attempt to activate lexical signs (Gutierrez-Sigut, Daws, et al., 2015, Marshall et al., 2014). However, we did not experimentally manipulate the amount of overt motor movement required.

In the current study we test the hypothesis that strength of lateralization increases with overt motor movement by directly comparing laterality indices across covert and overt sign generation tasks. These data are then compared to previously reported data from hearing native speakers of English who did not know BSL (Gutierrez-Sigut, Payne, & MacSweeney, 2015) and from hearing bimodal bilinguals (native users of BSL and English; Gutierrez-Sigut, Daws, et al., 2015) performing the same covert and overt tasks in English. Crucially, we contrast sign and speech LIs during *covert* language production, when no overt motor movement is required during the recording period. A finding of stronger lateralization for BSL than English generation in the covert tasks, would suggest that explicit motor movement does not make a major contribution to the strength of lateralization observed during overt sign production. The direct comparison of covert and overt tasks also allows assessment of the impact of continuous body movements on the quality of the TCD signal. Finding a similar number of unusable trials due to artefacts in both tasks would contribute to the development of strong experimental paradigms to assess the factors influencing lateralization during online language production.

Another possible explanation for the previously observed elevated LIs during sign compared to speech production (Gutierrez-Sigut, Daws, et al., 2015) is the language dominance of the participants tested. Participants in our previous study were hearing native signers. Although these individuals have deaf parents and have learned BSL from birth, their main means of communication and dominant language is English, reflecting the dominant language of the majority community, (see Emmorey, Giezen, & Gollan, 2015). It is possible that they found the tasks more challenging than deaf native signers (Emmorey et al., 2013, Emmorey et al., 2015). Certain aspects of task difficulty can influence the strength of lateralization as measured with fTCD (Payne, Gutierrez-Sigut, Subik, Woll, & MacSweeney, 2015). This raises the possibility that the elevated LIs were due to generating lexical items in their less dominant language, which makes the task more challenging. Additionally, phonological fluency has been shown to be more challenging for signers than semantic fluency (see Marshall et al., 2014 for a discussion). Here we examine the strength of lateralization during BSL phonological and semantic fluency tasks and its relationship with behavioural measures in a group of deaf native signers, whose dominant language is BSL. We predicted similar levels of lateralization between phonological and semantic signed tasks although the phonological overt condition was expected to be less productive (see Gutierrez-Sigut, Daws, et al., 2015, Marshall et al., 2014). Furthermore, elevated LIs for deaf native signers producing signs than for native English speakers producing speech, during both the semantic and phonological fluency tasks, would support the idea that the stronger left lateralization shown for hearing native signers producing BSL is not due to the difficulty of performing a task in a less dominant language.

## 2 Methods

### 2.1 Design

A 2 (*production type*: covert vs. overt) × 2 (*task:* phonological vs. semantic) design was used, resulting in four conditions: phonological-covert, phonological-overt, semantic-covert and semantic-overt. In the English phonological task,^2^ a series of letters are displayed and participants are asked to generate words beginning with this letter. In contrast, in our BSL phonological task participants are asked to generate signs containing a particular handshape (a major phonological parameter of signs). Here we use the term phonological fluency to refer to the analogous tasks in both languages for clarity and comparability with previous results. The semantic task proceeds in the same way, but here the cue is a semantic category.

These four conditions were presented in separate blocks, the order of which was counterbalanced across participants. Data from the deaf participants, who completed the tasks in BSL, were compared to two previously published datasets. One from hearing non-signers (Gutierrez-Sigut, Payne, et al., 2015) and one from hearing native signers (Gutierrez-Sigut, Daws, et al., 2015) who performed the same tasks in English.

### 2.2 Participants

Sixteen deaf native signers of BSL (9 female) were recruited from a volunteer database. The mean age of participants was 26 (*SD* = 5.9 range 16.9–36). All participants were profoundly deaf from birth and learnted BSL as their first language from their deaf parents. No participants reported a history of neurological disorders or language related problems. Participants were all right handed as assessed by the abridged version of the Edinburgh Handedness Inventory (Oldfield, 1971). Since all participants were signers, handedness for sign production was also assessed. Participants were asked to produce nine signs (all of which are produced in BSL with the dominant hand alone in BSL), to count to 20 (the dominant hand is always used) and to fingerspell three items (the dominant hand is clearly evident from fingerspelling production; see Sharma, 2013).

Due to insonation difficulties it was not possible to find the TCD signal in one participant. Of the 15 remaining participants (8 female; mean age 26.4, *SD* = 6.1, range 16.9–36), it was not possible to acquire a reliable TCD signal in one or more of the four conditions in four participants: one had poor data for both covert conditions (see Fig. 2 panel a: orange diamond), one for the semantic covert (see Fig. 2 panel a: green dash), one for the phonological overt (see Fig. 2 panel a: red square) and one for the semantic overt (see Fig. 2 panel a: blue triangle). Eleven participants had good quality data for all four conditions. Participants without TCD data in all four conditions were not included on the repeated measures ANOVAs. However, data from these participants in conditions where they had good signal were included in the correlational analyses with behavioural measures.^3^

In a previously published fTCD study we tested 22 hearing non-signing participants (8 female) on English versions of the four experimental conditions tested here in BSL (Gutierrez-Sigut, Payne, et al., 2015). To compare hemispheric lateralization during sign and speech production, the current data were contrasted with the data from the previous study. The mean age of the hearing participants was 27.2 (range 19–46, *SD* = 6.3). All were right–handed, had English as their first language and had no knowledge of BSL. No participants reported a history of neurological disorders or language related problems.

### 2.3 Stimuli

**Fig. 1:**
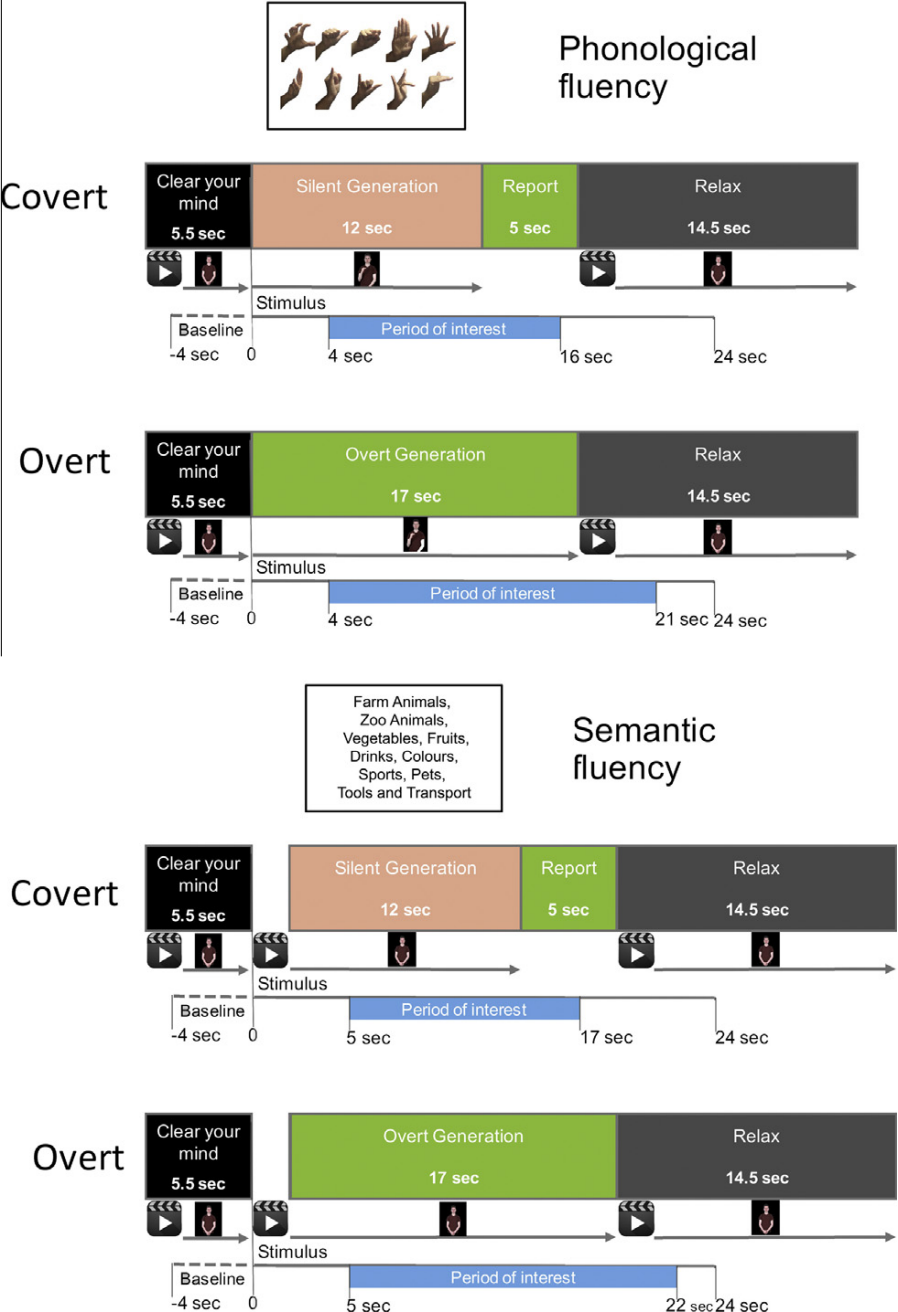
Diagram of selected stimuli, timings of procedure and timings of fTCD data entered in the analyses for each of the experimental conditions.

*BSL phonological fluency task* – 10 BSL handshapes were chosen which have been used in a previous BSL phonological fluency study (see Gutierrez-Sigut, Daws, et al., 2015). Each handshape was presented twice within each condition: covert phonological fluency/overt phonological fluency. Thus, each condition consisted of 20 trials, which were presented in a pseudo-randomized order. Fig. 1 (top) shows the selected handshapes.

*BSL semantic fluency task* – the following 10 categories were chosen: Farm Animals, Zoo Animals, Vegetables, Fruits, Drinks, Colours, Sports, Pets, Tools and Transport. These categories were repeated twice within each of the semantic fluency task blocks, resulting in 20 trials per block, which were presented in a pseudo-randomized order (as above).

In our previous studies of *English* phonological fluency in hearing adults, participants named as many words as possible starting with a target letter, presented visually. Ten letters (A, B, C, F, H, M, O, S, T and W) were presented twice throughout the 20 trials (see Gutierrez-Sigut, Payne, et al., 2015 for details). The same semantic categories were used as in the current BSL study.

### 2.4 Procedure

Ethical approval for the study was obtained from the UCL Research Ethics Committee. All participants gave written informed consent prior to the study. The whole session, including setup time, lasted approximately 120 min. Each block was preceded by two practice trials showing categories or handshapes that were not used in the experimental blocks.

#### 2.4.1 Covert generation

Each trial began with a 5.5 s preparation period during which the participants were instructed to focus on the screen. A BSL ‘clear your mind’ video was displayed, with the last frame frozen on the screen, was shown for the remaining time of the preparation period (see Fig. 1).

In the phonological block, a still image showing the target handshape was displayed for 12 s. Participants were required to silently generate as many signs as possible that included the target handshape. In the semantic block a video clip of the BSL sign for the semantic category was displayed. The last frame of the sign remained on screen for 12 s. Participants were required to silently generate as many signs as possible belonging to the target category. To ensure compliance with the task, at the end of the covert phase participants were asked to overtly report as many of the signs they had generated as possible. This ‘report’ period lasted for five seconds. The report phase was followed by a ‘relax’ phase (14.5 s) in which participants were asked to imagine a ‘peaceful’ scene. The ‘relax’ prompt was presented in BSL. The overall trial duration was 37 s for the phonological block and 38 s for the semantic block. Note that the semantic block is longer to allow time for participants to see the video clip of the category prompt and then have the same amount of generation time as in the phonological condition. The prompt in the phonological condition was a static image that remained on the screen throughout the generation phase.

#### 2.4.2 Overt generation

The overt blocks proceeded in the same way as the covert blocks, except that the participants reported the signs as soon as the stimulus had been presented. The generation period was 17 s.

In the covert English tasks, performed by the hearing participants, the trial sequence was as follows: 3 s ‘clear your mind’ period; 12 s silent generation period; 5 s ‘report’ period; 10 s ‘relax’ period. The overt tasks proceeded in the same way except that the participants reported the words as soon as the stimulus was displayed. This generation period lasted 17 s (see Gutierrez-Sigut, Payne, et al., 2015 for details).

Stimuli were presented using Cogent toolbox (www.vislab.ucl.ac.uk/cogent) for MATLAB (Mathworks Inc., Sherborn, MA, USA). Triggers time-locked to the onset of the stimulus were sent from the presentation PC to the Doppler-Box set-up.

### 2.5 Behavioural data scoring and video coding

Participant’s behavioural responses were monitored on-line and were video recorded for scoring offline. The number of lexical signs produced in each trial was counted. In order to explore the effect of arm and hand movement during the overt generation tasks on the TCD signal, participant’s movements during the generation periods were coded, offline, by a deaf BSL signer. All movements produced during the generation period were coded using three categories (1) the participant made a one-handed sign moving only the right hand, (2) the participant made a two-handed sign in which the right hand was dominant and (3) the participant made a two-handed sign in which both hands moved symmetrically. The amount of time (in seconds) spent on each of these movements was calculated. Movement of the left hand alone accounted only for an average of 1.2% of the total session time and was thus not coded further.

### 2.6 fTCD recording and processing

Blood flow velocity through the left and right MCAs was examined using a Doppler ultrasonography device (DWL DopplerBox: manufactured by DWL Elektronische Systeme, Singen, Germany). Two 2-MHz transducer probes were mounted on a flexible headset and placed at each temporal skull window.

Data analysis was carried out with dopOSCCI, a custom MATLAB (Mathworks Inc., Sherborn, AM, USA) program written for analysing fTCD group data (Badcock, Holt, Holden, & Bishop, 2012). Analysis involved down-sampling of the data from 100 to 25 Hz, normalization of left and right channel values, heart cycle integration and artefact rejection. Epochs with values less than 70% or greater than 120% of the average blood flow velocity were excluded from the analyses. Epochs were segmented from – 4 to 24 s relative to stimulus presentation. All data points were baseline corrected by subtracting the blood flow velocity during a period of inactivity – 4 to 0 s prior to stimulus onset. To ensure that blood flow for the baseline period was always calculated from resting level, the first trial of the block was not included in the analyses. This resulted in 19 analysed trials per block.

In order to accurately capture blood flow velocity changes related to sign generation the period of interest (POI) was set independently for each of the experimental conditions. For the covert phonological condition the POI was set from 4 to 16 s, so that blood flow changes due to overt production during the report period are not likely to be included in the calculations. For the overt phonological condition the POI was 4–21 s, including the whole generation period. For the semantic blocks the POIs were delayed by 1 s to account for the time required to see the sign’s video clip before start signing. POI was setup to 5–17 s for the covert and 5–22 s for the overt block. Laterality indices (LIs) were calculated for each participant separately, for each of the four conditions. For each participant the maximum peak left-right difference within the POI was identified. A two second window was centred on this maximum. The LI was defined as the average of the left minus right differences within this two second window. Data from the English study were analysed in the same way. The baseline was set from −8 to 4 s before stimulus onset and POI was set from 4 to 14 s after stimulus onset to maximize the likelihood that the blood velocity changes due to linguistic processing were captured (see Gutierrez-Sigut, Payne, et al., 2015 for details).

One-sample *t*-tests were used to assess whether the LI value was significantly left or right lateralized for each participant in each condition. When the one-sample *t*-test did not reach significance, participants were considered ‘low lateralized’.

## 3 Results

### 3.1 fTCD data quality and reliability

The average number of accepted epochs across conditions was 15 (*SD* = 2.2, min = 8, max = 19; Table 1 shows the mean number of accepted trials for each condition). To investigate whether overt signing led to more movement artefacts in the fTCD data than covert signing we analysed the number of epochs remaining after artefact rejection (see Section 2). A repeated measures ANOVA revealed no differences in the number of epochs accepted between tasks [F(1, 10) = 1.52, MSE = 5.99, *p* = 0.246, η_p_^2^ = 0.132], or production types [F(1, 10) < 1] and there was no significant interaction [F(1, 10) < 1]. Furthermore, the standard deviations of the epoch LIs for individuals suggested that variability in LIs was similar across overt and covert conditions: phonological-covert (mean = 3.6, range: 6.5–2.1), phonological-overt (mean = 4.1, range: 5.9–2.7), semantic-covert (mean = 3.9, range: 6.1–2.5) and the semantic-overt (mean = 3.7, range: 6.8–1.9).

Split half reliability analyses were conducted on each condition separately. Good reliability (correlation between LIs in odd and even epochs) was observed in the phonological-covert (r = 0.54, *p* = 0.045), phonological-overt (r = 0.66, *p* = 0.011) and the semantic-overt (r = 0.61, *p* = 0.021) conditions, but not the semantic-covert (r = −0.19, *p* = 0.535).

#### 3.1.1 Contrasting BSL and English data: Impact of sign and speech generation on data quality

To test the hypothesis that movement due to sign language production might lead to more rejected epochs due to artefacts than speech production we compared the present data with the previously published data from hearing participants performing the same covert and overt tasks in English (Gutierrez-Sigut, Payne, et al., 2015). We carried out a mixed ANOVA on the number of rejected trials with the factor *language* (BSL vs. English) as a between subjects factor and experimental *task* (phonological-covert, phonological-overt, semantic-covert and semantic-overt) as a within subjects factor. There were no significant effects of *task* [F(3, 93) = 1.6, MSE = 5.4, *p* = 0.288, η_p_^2^ = 0.5], or *language* [F(1, 31) < 1] and no interaction [F(3, 93) < 1].

### 3.2 Mean Laterality Index (LI) and percentage of deaf participants left lateralized

**Fig. 2:**
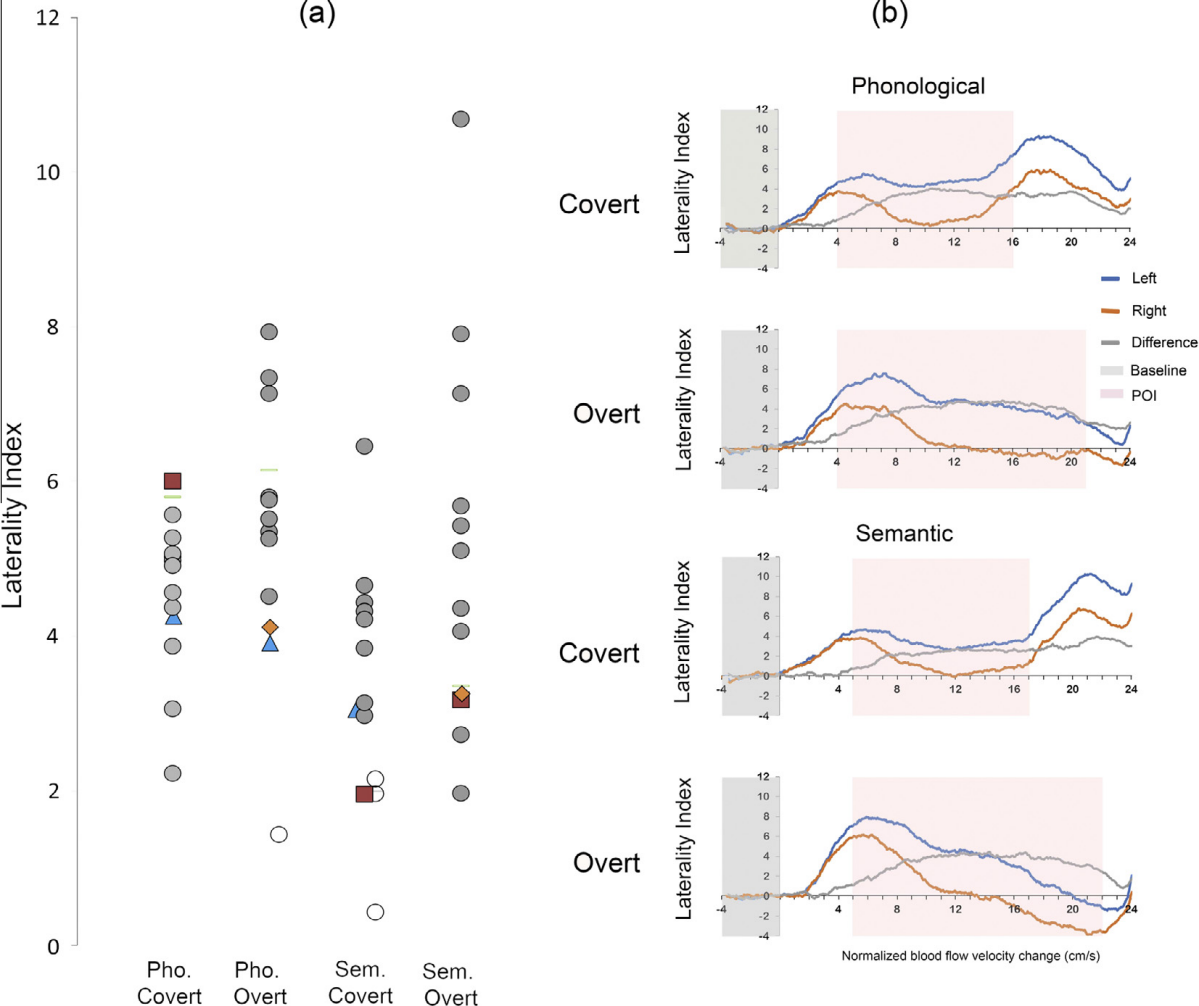
Panel (a) shows a scatterplot of the individual LIs for each of the BSL generation conditions. White circles show participants classified as low laterality in this condition. The three participants with data missing from one or more conditions, who are therefore removed from the repeated measures analysis, are shape-coded and are presented in shapes other than circles. Panel (b) shows the group level average of the baseline-corrected cerebral blood flow velocity for each of the conditions for the left (blue line) and right (red line) channels as well as the difference (left minus right; grey line). The grey section depicts the baseline and the pink section depicts the POI within which the LIs were calculated from the individuals’ maximum left-right difference.

At the group level, each of the four conditions BSL conditions was significantly left lateralized (see Table 1 and Fig. 2). Table 1 shows the results of the one-sample *t*-tests at the group level as well as the percentages of participants who showed left lateralization and low laterality (not significantly different to zero) in each condition. None of the participants showed a negative LI.

### 3.3 Laterality Index (LI) differences between BSL conditions

**Fig. 3:**
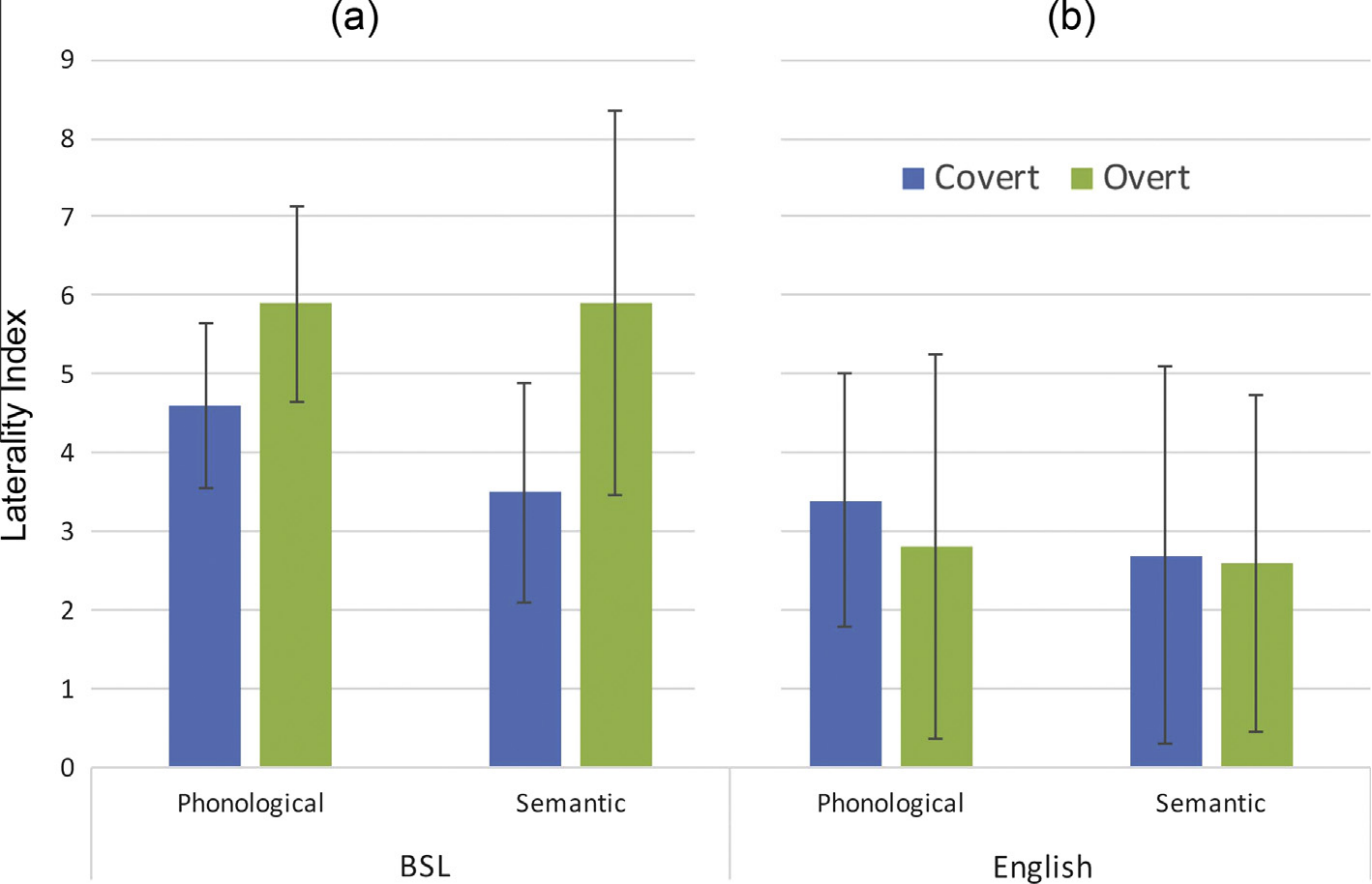
Mean LI summaries for phonological and semantic fluency in the covert (blue bar) and overt (green bar) conditions. Panel (a) shows mean LIs for the BSL generation tasks. Panel (b) depicts mean LIs for the previously collected English generation data (Gutierrez-Sigut, Payne, et al., 2015). Error bars represent standard deviation.

A repeated measures ANOVA of the LIs showed a main effect of *production type* [F(1, 10) = 8.8, MSE = 3.14, *p* = 0.014, η_p_^2^ = 0.468] indicating that the covert tasks generated lower LIs than overt tasks (mean 3.9 vs. 5.5). The main effect of *task* (phonological versus semantic) [F(1, 10) = 1.16, MSE = 2.7 *p* = 0.306, η_p_^2^ = 0.104] and the interaction [F(1, 10) = 1.8, MSE = 2.07 *p* = 0.205, η_p_^2^ = 0.155] were not significant. Fig. 3 (panel a) shows the LIs for each of the four conditions.

#### *3.3.1* *Contrasting BSL and English data:* Comparing LIs during English and BSL generation

To compare hemispheric lateralization during sign and speech production, the current data were contrasted with the previously published data from hearing participants performing the tasks in English (Gutierrez-Sigut, Payne, et al., 2015). A mixed model ANOVA was used, including the between subject factor *language* (BSL vs. English) and the within subject factors *production type* (overt vs. covert) and *task* (phonological vs. semantic). There was a significant main effect of *language* [F(1, 31) = 13.2, MSE = 7.4 *p* = 0.001, η_p_^2^ = 0.299]. This indicated larger LIs for BSL than English generation (means of 4.7 vs. 2.9). The main effect of task just failed to reach significance [F(1, 31) = 3.7, MSE = 1.9, *p* = 0.064, η_p_^2^ = 0.106] (phonological mean LI = 4.1, semantic mean LI = 3.6). The main effect of production type [F(1, 31) = 5.2, MSE = 5.33, *p* = 0.165, η_p_^2^ = 0.061] was not significant.

There was a significant interaction between production type and language [F(1, 31) = 5.3, MSE = 5.33, *p* = 0.029, η_p_^2^ = 0.145]. Pairwise comparisons showed no differences between covert and overt conditions for the English generation (mean 3.1 vs. 2.7 [F(1, 31) < 1]) but a significant difference in BSL generation [F(1, 31) = 5.2, *p* = 0.005], with lower LIs for the covert than the overt conditions (mean 3.9 vs. 5.5). The interaction between task and language [F(1, 30) < 1], as well as the three-way interaction [F(1, 30) < 1], were not significant.

In order to exclude the possibility that stronger LIs found during BSL generation in deaf participants than English generation in hearing non-signers were due to the difference in bilingual status of the participants (all deaf signers are bilingual to some extent (for a commentary see Woll & MacSweeney, 2015), we used another set of previously published data from *hearing native signers* (Gutierrez-Sigut, Daws, et al., 2015). LIs measured during the overt tasks from deaf signers producing BSL were compared to those from hearing native signers performing the tasks in English. A 2 × 2 mixed ANOVA including the between subject factor *group* (deaf signers vs. hearing signers) and the within subject factor *task* (phonological vs. semantic) showed a significant main effect of *group* [F(2, 27) = 22.3, MSE = 6.08, *p* < 0.001, η_p_^2^ = 0.452]. LIs were lower for the hearing native signers performing the task in English than for the deaf signers performing the task in BSL (mean 2.3 vs. 5.5). The main effect of *task* [F(1, 27) < 1] and the interaction [F(1, 27) < 1] were not significant.

For completeness we also contrasted LIs from deaf and hearing native signers performing the overt tasks in BSL (from Gutierrez-Sigut, Daws, et al., 2015). The main effects of *group* [F(1, 27) < 1] and *task* [F(1, 27) < 1] as well as the interaction [F(1, 27) < 1] were not significant.

### 3.4 Behavioural data

#### 3.4.1 Number of items produced during BSL overt fluency task and correlations with LI

A paired sample *t*-test showed that participants produced more signs in the semantic than in the phonological task (mean of 5.7 vs. 9.7, t(12) = −8.54, *p* < 0.001).

However, strength of LI did not correlate with the number of signs produced in the phonological overt (r = −0.136, *p* = 0.642) or semantic overt (r = −0.413, *p* = 0.142) conditions.

##### 3.4.1.1 Contrasting BSL and English data: Number of items generated

We compared the number of signs produced in the overt tasks by the deaf participants to the number of English words produced by hearing non-signing participants. A mixed ANOVA with the between participants factor *language* (BSL vs. English) and within participants factor *task* (phonological vs. semantic) showed a main effect of language [F(1, 33) = 4.73, MSE = 5.26 p = 0.037, η_p_^2^ = 0.125], a main effect of task [F (1, 33) = 61.1, MSE = 1.59, *p* < 0.001, η_p_^2^ = 0.649] and a significant interaction [F(1, 33) = 26.2, MSE = 1.59, p < 0.001, η_p_^2^ = 0.443]. More items were produced in both languages in the semantic than in the phonological task; BSL [F(1, 33) = 66.56, p < 0.001] and English [F(1, 33) = 4.89, p = 0.034]. Pairwise comparisons showed that there were no differences between the two languages in the number of items produced in the semantic task [F(1, 33) < 1] but fewer items were produced in the BSL than in the English phonological task [F(1, 33) = 26.65, p < 0.001].

For completeness we also contrasted the number of signs produced by deaf and hearing native signers performing the overt tasks in BSL (from Gutierrez-Sigut, Daws, et al., 2015). There were significant main effects of *group* [F(1, 27) = 25.56, MSE = 2.20, p < 0.001, η_p_^2^ = 0.486] and *task* [F(1, 27) = 150.42, MSE = 1.25, p < 0.001, η_p_^2^ = 0.848]. The interaction was not significant [F(1, 27) = 1.98, MSE = 1.25, p = 0.171, η_p_^2^ = 0.068]. More items were produced in the semantic than in the phonological task (phonological: mean = 4.8, *SD* = 1.3; semantic: mean = 8.4, *SD* = 1.97). The deaf signers produced more than the hearing signers (deaf: mean = 7.7, *SD* = 1.3; hearing: mean = 5.7, *SD* = 1.2).

#### 3.4.2 Handedness of movements produced during BSL overt fluency tasks

**Fig. 4:**
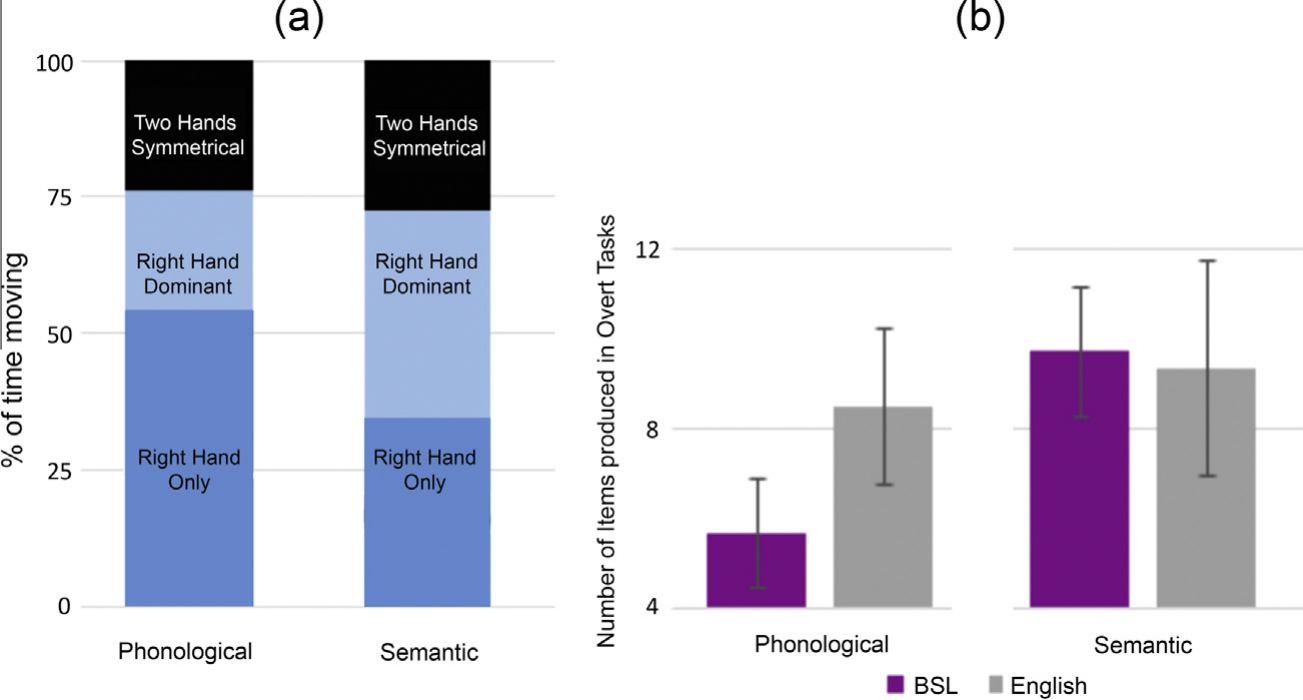
Panel (a) shows the mean percentage of time spent producing each type of movement during the overt generation trials for the phonological and semantic conditions. Panel (b) shows the number of lexical items produced in the BSL (purple bars) and English (grey bars; see Gutierrez-Sigut, Payne, et al., 2015) overt generation tasks. Error bars represent standard deviation.

To examine the relationship between hand movement and LI we undertook detailed analysis of the movements produced in the overt tasks (see Section 2). A repeated measures ANOVA with the factors *task* (phonological vs. semantic) and *sign type* (right hand only/right hand dominant/two hands symmetrical) showed a significant main effect of *task* [F(1, 12) = 19.9, MSE = 0.543, *p* = 0.001, η_p_^2^ = 0.623], indicating that participants spent less time moving the hands during the phonological than the semantic task (means of 5.4 vs. 6.1 s per trial). There was a main effect of *sign type* [F(2, 24) = 13.2, MSE = 5.14, *p* < 0.001, ηp^2^ = 0.524], indicating that more time during each trial was spent producing right hand only movements (mean = 7.6 s) than either of the other two movement classifications: two-handed right hand dominant movements (mean = 5.3 s) and two-handed symmetrical movements (mean = 4.5 s). There was also a significant interaction [F(2, 24) = 11.997, MSE = 4.96, *p* < 0.001, η_p_^2^ = 0.500]. Pairwise comparisons showed that more time was spent producing right hand only movements in the phonological than in the semantic fluency task (means 8.8 and 6.3 s respectively; [F(1, 12) = 8.92, *p* = 0.011]). Conversely, more time was spent during the semantic than during the phonological task producing right hand dominant (means 7.01 vs. 3.5 s; [F(1, 12) = 20.68, *p* = 0.001] and two-handed symmetrical movements (mean 5.1 vs. 3.8 s [F(1, 12) = 4.8, *p* = 0.049]) (see Fig. 4).

### 3.5 Correlations with LI

Strength of LI during the BSL phonological task and the BSL semantic task did not correlate with amount of time producing movements during the trial (coded into three different classifications): right hand only movements (phonological - r = −0.36, *p* = 0.208; semantic - r = 0.18, *p* = 0.542), right hand dominant (phonological r = 0.06, *p* = 0.837; semantic - r = −0.12, *p* = 0.690) or two hands symmetrical movements (phonological r = −0.17, *p* = 0.557; semantic r = 0.04, *p* = 0.886).

To explore more thoroughly the effects of dominant hand movement, we combined the measures of movement in the right hand only and right hand dominant conditions. There was no correlation between strength of LI and this movement measure during either the phonological (r = −0.33, *p* = 0.251) or semantic task (r = 0.01, *p* = 0.555).

## 4 Discussion

The aim of this study was to examine the possible causes of the previously observed elevated LIs for BSL compared to spoken English production in hearing native signers (Gutierrez-Sigut, Daws, et al., 2015). We addressed the contribution of motor movement by directly comparing overt and covert BSL generation. We also addressed whether language dominance (of English) might contribute to this effect in hearing native signers, by testing deaf native signers, dominant in BSL.

The mean LI for all participants was positive and the majority were classified as left lateralized for all conditions. LIs were stronger for the overt than the covert conditions, suggesting some contribution of explicit movement to the LIs. However, the strength of lateralization was not correlated with the amount of time moving the right hand in any of the overt tasks. The comparison with LIs of hearing speakers performing the same tasks in English showed that lateralization was stronger for BSL production in all conditions, including the covert tasks in which no movement was required during the recording period. These findings suggest that explicit motor movement makes only a limited contribution to the strength of lateralization during sign production as measured with fTCD. That LIs were greater for deaf signers producing BSL than hearing participants producing English, suggests that language dominance cannot explain the enhanced LIs observed for BSL production in our previous study with hearing native signers (Gutierrez-Sigut, Daws, et al., 2015). We discuss the contribution of these two potential contributing factors further below.

### 4.1 The role of motor movement

Unlike the hearing participants producing speech, deaf native signers showed stronger left lateralization for (BSL) production in the overt than the covert conditions. In contrast to speech production, overt sign production requires precise movements of large body areas, particularly of the arms and hands. Signers are dominant in the use of one hand (Vaid, Bellugi, & Poizner, 1989). Some signs are produced with the dominant hand alone, whilst two-handed signs can either be symmetrical or, in most cases, asymmetrical – with the dominant hand producing the majority of the movement (see Battison, 1978). Our current finding reveals that this asymmetric movement during overt signing contributes, at least to some extent, to the observed patterns of hemispheric lateralization in deaf native signers. This is in line with results from Emmorey et al. (2014) who used H_2_^15^O-PET to directly contrast ASL and English production during picture naming in signers, without removing low-level motoric effects. An increased left posterior parietal activation found in signers was linked to the voluntary production of motor movements.

Importantly, LIs of deaf signers during BSL generation was stronger than those for English speakers during speech generation, not only for the overt but also for the covert tasks. This replicates our previous finding of a stronger left lateralization for BSL than for speech overt production in hearing native signers (Gutierrez-Sigut, Daws, et al., 2015). Furthermore, the fact that LIs are stronger for BSL than for English in the covert generation conditions shows that explicit motor movement, although a contributing factor, is not the primary driver of the increased LIs during sign production. An explicit test of this hypothesis would be to ask right handed participants to produce signs with their left hand (only 1.2% of signs in the current dataset were produced with the left hand alone). Based on the positron emission tomography (PET) study by Corina and colleagues (Corina et al., 2003) we would predict left hemispheric lateralization under these conditions. However, it would be interesting to address whether the LIs measured using fTCD were weaker for left handed than right handed sign production in right handers. A further unanswered question is whether different types of phonological movement affect the LIs differently. For example, it is possible that hand internal movements affect the LI differently than path movements or that phonological movements show a larger effect on the LIs than transitional movements.

In the current study the strength of lateralization did not correlate with extent of movement of the right hand during either the phonological or the semantic overt fluency tasks. This is consistent with our previous data from hearing native signers (Gutierrez-Sigut, Daws, et al., 2015) performing the BSL semantic fluency task. However, hearing signers *did* show a moderate correlation between strength of LI and movement of the right hand during the phonological task. One possible reason for this apparent discrepancy is that hearing signers found the phonological task more difficult than deaf signers. Indeed, they produced fewer signs overall. Due to difficulty of the task the hearing signers may have used a wider set of strategies than the deaf to guide their phonological search. These strategies could include more extensive movement of the dominant hand while holding the cued handshape as well as silent labelling of items using English phonology. It is possible that the combination of these factors led to a different relationship between LIs and hand movement measures in the signing groups.

Our results add to the neuroimaging literature that has linked factors such as increased proprioceptive monitoring and the special nature of phonological encoding of signs with greater left parietal lobe activation for sign than speech production (Braun et al., 2001, Corina et al., 2003, Emmorey et al., 2007). For example, Emmorey et al. (2007) used PET to compare brain activation of deaf native signers naming objects in American Sign Language (ASL) and hearing speakers naming in English. Results showed similar activation in classical language areas for both ASL and English. Furthermore, there was greater activation in the left parietal lobe for sign production that was attributed to proprioceptive monitoring and phonological encoding of signs (see also Braun et al., 2001, Emmorey et al., 2014).

Additionally, our results show that overt sign generation did not result in an increased number of rejected trials nor in a lower internal reliability due to poorer signal quality than covert sign generation. When compared with data from the same tasks in English, the quality of the signal was also similar. Together with our previous data (Gutierrez-Sigut, Daws, et al., 2015) it seems clear that continuous movement of the arms and hands does not disrupt the assessment of hemispheric lateralization as measured with fTCD. This highlights the feasibility of fTCD as a reliable way of measuring hemispheric dominance during natural language processing, allowing the use of more naturalistic experimental settings where participants can produce fully formed signs, as opposed to the ‘whispered signs’ often required in fMRI studies of sign language production (Emmorey et al., 2007, Emmorey et al., 2014). The findings also support the use of fTCD as a tool to examine, hemispheric dominance during cognitive tasks with children who use a signed language and those with cochlear implants. The use of overt signing also allows for strict control over the output produced by the participant, allowing a more accurate assessment of the factors that influence variations in hemispheric dominance. In the current study, as in the case of hearing native signers (Gutierrez-Sigut, Daws, et al., 2015), no correlation was found between strength of laterality and number of signs produced in the overt conditions. This contrasts with results from English speakers performing the tasks in English, where a positive correlation has been found (Gutierrez-Sigut, Daws, et al., 2015, Gutierrez-Sigut, Payne, et al., 2015). Further research is needed to fully explore these relationships between LIs and behaviour both in speakers and signers.

### 4.2 The role of language dominance and task difficulty

Laterality indices were higher for deaf native signers performing the task in BSL than for both native English speakers and hearing native signers performing the task in English. However, LIs did not differ between deaf and hearing signers performing the tasks in BSL. These data indicate that task difficulty due to performing the task in a less dominant language does not account for the previously reported increase in LIs in hearing native signers during sign generation. Additionally, the overall strength of LIs in deaf native signers did not differ between phonological and semantic sign generation either in the overt or the covert conditions. These results are consistent with previous fTCD findings both in native English speakers and hearing native signers (Gutierrez-Sigut, Payne, et al., 2015, Gutierrez-Sigut, Daws, et al., 2015) and contribute to the characterization of which aspects of task difficulty affect the fTCD signal. Our finding is in line with previous results in which difficulty of phonological search in a word generation paradigm did not result in a difference of LIs although participants produced more items in the easy than the difficult condition (Badcock et al., 2012, Dräger and Knecht, 2002).

In accordance with previous findings from deaf native signers (Marshall et al., 2014), hearing native signers (Gutierrez-Sigut, Daws, et al., 2015) and speakers (Crowe, 1998, Hurks et al., 2006, Monsch et al., 1994) participants produced more items during the semantic than the phonological fluency task. Notably, combining these data with those of native English speakers showed that although both groups produced more in the semantic tasks, the deaf signers produced a remarkably low number of signs in the phonological task. This finding, together with data from the amount of time producing movements of the right hand alone, which was larger for the phonological than for the semantic task, indicates that participants might be adopting a different strategy for BSL phonological fluency. As in previous studies of sign production (Gutierrez-Sigut, Daws, et al., 2015, Marshall et al., 2014), deaf native signers often rehearsed several movements in a range of locations while holding the cued handshape in their hand. The use of the phonological fluency task (also referred to as a Word Generation task) has provided remarkably consistent results for assessing lateralization of speech with fTCD, consequently becoming the gold standard. The present findings indicate that phonological fluency can be reliably used to assess lateralization for language in signers. However, as in our previous study, differences in behavioural results reveal the importance of taking a multidimensional approach by studying different language subdomains. This can be particularly relevant for the study of sign generation and how linguistic processes are affected by modality.

Although research into sign production is still in its infancy, there are indications that results from signed and spoken phonological fluency tasks might not be directly comparable (Gutierrez-Sigut, Daws, et al., 2015, Marshall et al., 2014). First, tasks demands are not identical. While in the signed phonological task participants are presented with a major phonological parameter (handshape) in the English task the cue for word generation is an orthographic representation of the target phoneme (letter). Second, the phonological fluency task in BSL might be more influenced by strategic and non-linguistic factors than the English task. Marshall et al. (2014) reported data from a behavioural study of phonological and semantic fluency in deaf native BSL users. They found the expected similarities with speech production for the semantic fluency task. However, responses to phonological categories were less productive in the signers than they typically are in speakers. Furthermore, analysis of the types of clustering within categories revealed a close relationship between semantics and phonology in the signs generated. The present behavioural findings add to the proposal that an overt semantic fluency task might be a more appropriate task to assess sign language generation processes.

## 5 Conclusions

Like speech, sign language production appears to rely primarily on a left lateralized network (Bellugi et al., 1988, Corina, 1999, Corina et al., 1999, Damasio et al., 1986, MacSweeney, Capek, et al., 2008, MacSweeney, Waters, et al., 2008). The current study of BSL generation in deaf native signers shows stronger left hemisphere lateralization than in hearing speakers performing the task in English. This supports our previous data from hearing native signers and clarifies that our previous findings, with hearing signers, cannot be explained by the fact that hearing native signers were performing the tasks in a less dominant language or be wholly attributed to the motor movement. Importantly, left lateralization of signers was stronger than that of speakers in the overt and in the covert tasks. This finding shows that factors specific to signing, other than continuous movement of the arms and hands, are the main contributors to the increased left lateralization found for sign production in both deaf (present study) and hearing signers (Gutierrez-Sigut, Payne, et al., 2015). Fundamental differences between sign and speech in the nature of phonological encoding and the use of self-monitoring mechanisms may have important implications for lateralization of sign language production.

## Figures and Tables

**Table 1:** Mean LI and percentages of left lateralized signers.

Task	Production type	Accepted epochs	LI				Left lateralized	Low laterality
Mean (*SD*)	Mean	*SD*	t	p	% (#)	% (#)
Phonological	Covert	15.8 (2.3)	4.5	1.1	15.6	0.000	100 (14)	0
Overt	15.9 (2.6)	5.5	1.9	11.6	0.000	92.9 (13)	7.1 (1)

Semantic	Covert	15.4 (4)	3.4	1.4	9.2	0.000	76.9 (10)	23.1 (3)
Overt	14.6 (3.1)	5.5	2.6	7.7	0.000	100 (14)	0

## Footnotes

2 The phonological fluency task is often referred to in the fTCD literature as “Word Generation”.

3 The exclusion of participants with incomplete datasets from the correlational analyses did not alter the pattern of results.
